# S-nitrosylation of the thioredoxin-like domains of protein disulfide isomerase and its role in neurodegenerative conditions

**DOI:** 10.3389/fchem.2015.00027

**Published:** 2015-04-16

**Authors:** Myra E. Conway, Matthew Harris

**Affiliations:** Department of Applied Sciences, University of the West of EnglandBristol, UK

**Keywords:** PDI, thioredoxin-like -CXXC- motifs, protein folding, S-nitrosylation, neurodegeneration

## Abstract

Correct protein folding and inhibition of protein aggregation is facilitated by a cellular “quality control system” that engages a network of protein interactions including molecular chaperones and the ubiquitin proteasome system. Key chaperones involved in these regulatory mechanisms are the protein disulfide isomerases (PDI) and their homologs, predominantly expressed in the endoplasmic reticulum of most tissues. Redox changes that disrupt ER homeostasis can lead to modification of these enzymes or chaperones with the loss of their proposed neuroprotective role resulting in an increase in protein misfolding. Misfolded protein aggregates have been observed in several disease states and are considered to play a pivotal role in the pathogenesis of neurodegenerative conditions such as Alzheimer's disease, Parkinson's disease, and Amyotrophic Lateral sclerosis. This review will focus on the importance of the thioredoxin-like CGHC active site of PDI and how our understanding of this structural motif will play a key role in unraveling the pathogenic mechanisms that underpin these neurodegenerative conditions.

## Introduction

**Figure 1:**
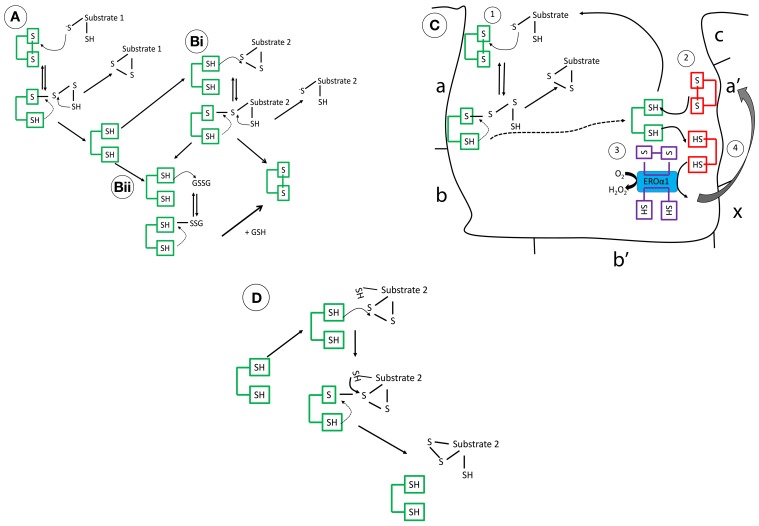
**The mechanistic details of PDI as (A) an oxidase (B) a reductase (C) interaction of reduced PDI with ERO1 (D) an isomerase**. These mechanisms are complex, governed in part by its intriguing structure but also the pKa of the involved cysteine residues and the redox microenvironment. **(A)** Operating as an oxidase, forming new disulfide bonds, PDI in its oxidized form, is targeted through the nucleophilic cysteine of the substrate, forming a mixed disulphide. The substrate is then released oxidized after donating its reducing equivalents to PDI. **(Bi)** As a reductase, the N-terminal cysteine of PDI, which has a low pKa (approximately 4.5), targets its substrate disulfide bond through a nucleophilic attack, forming a mixed disulfide. Now, the substrate thiol is free to interact with another disulfide bond. This results in the oxidation of PDI. **(Bii)** Alternatively, reduced PDI can be re-oxidized by GSSG or an oxidoreductase, the ER flavo-oxidase Ero1 (its preferred substrate), but peroxiredoxins are also efficient. The kinetic rates of reaction of PDI *in vitro* are dependent on the substrate and the GSH/GSSG ratio. **(C)** In brief, reduced PDI, generated after reaction 1(dotted line), is oxidized by the a′ domain (reaction 2), finally, reoxidation of the a′ domain is catalyzed by Ero1 and channels the electrons to final acceptors such as molecular oxygen (reaction 3/4) **(D)** Rearranging of disulfide bonds is less understood but incorrect disulfide bonding undergoes isomerization creating the correct pattern of disulfide bond formation.

Protein disulfide isomerases (EC 5.3.4.1) are key metabolic proteins which primarily reside in the endoplasmic reticulum (ER) and operate in synergy with other chaperones, such as glucose-regulated protein (78 kDa) (GRP78) that controls the processing and correct folding of proteins (Wilkinson and Gilbert, 2004). Expression of PDI has also been reported outside the ER, in the mitochondria, nucleus, cytosol and cell surface (Yoshimori et al., 1990; Koehler et al., 2006; El Hindy et al., 2014). PDI forms part of the thioredoxin superfamily, which catalyze the formation and breakage of disulfide bonds, through thiol:disulfide exchange, facilitating correct refolding of the protein by rearranging the configuration of disulfide bonds (Figure 1) (Tu et al., 2000). These proteins have also associated roles as chaperones in refolding without enzymatic involvement.

The functional diversity of the PDI family depends on its metabolic substrates, the redox environment, but more importantly their characteristic thioredoxin domain-like structures, which govern the redox thiol-disulfide isomerase and chaperone activity. Depletion of PDI in mammalian cells results in a delay in disulfide bond formation of secretory proteins, highlighting its vital role in oxidative protein folding (Rutkevich et al., 2010). Neurodegenerative diseases, including Alzheimer's disease (AD), Parkinson's disease (PD), and Amyotrophic lateral sclerosis (ALS) share several common pathological characteristics, in particular, the abnormal accumulation of misfolded proteins forming inclusions, where a common role for PDI has been assigned (Kim et al., 2000; Muchowski, 2002; Tuite and Melki, 2007). This review will focus on the function of PDI in protein folding and the impact of increased cellular stress on its proposed neuroprotective role.

## Structure of human PDI dictates its functional diversity

The PDI family has 21 members, all of which have thioredoxin-like fold structures, with most including 1–3 redox-active -CXXC- motifs (Ellgaard and Ruddock, 2005; Appenzeller-Herzog and Ellgard, 2008; Hatahet and Ruddock, 2009). The individual domains of human PDI [Figure 1C (**a**, **a′**, **b**, and **b**′)] have been resolved using NMR (PDB codes; 1Mek, 1X5C, 3BJ5, and 2K18) (Kemmink et al., 1996, 1999; Denisov et al., 2007; Nguyen et al., 2008). However, it was not until the recent crystallization of the three-dimensional structure of yeast PDI [4°C (PDB:2B5E) and 22°C (PDB:3BOA)] and human PDI (hPDI) [oxidized (PDB:4EL1) and reduced (PDB:4EKZ)] that insight into how these domains operate cooperatively was detailed (Tian et al., 2006; Wang et al., 2013). hPDI has four thioredoxin-like domains (**a**, **a′**, **b**, and **b′**), where two of these (**a** and **a′**) have a catalytic redox-active –CGHC- motif (Wang et al., 2013). In yeast (the structure resolved at 4°C) and hPDI, the **a**/**a′** domains face each other in a “U-shaped” structure and are solvent exposed allowing interaction with their respective substrates (Tian et al., 2006; Wang et al., 2013). The **b** domains confer substrate specificity and form a hydrophobic rigid base connecting the two flexible **a** domains. The **a′** and **b′** domain are connected through a 19 amino acid inter-domain link, called the x-linker, which is predicted to promote flexibility, influencing substrate binding (Wang et al., 2010). Another key feature is the highly-acidic C-terminus, which is involved in calcium binding and contains the ER-retrieval motif KDEL. In hPDI, the folds of the domains show conformational differences in the reduced and oxidized form, where the distance between the two active site -CXXC- regions increases from 27.6 to 40.3 Å, offering a more open structure exposing the hydrophobic areas of the b domains when oxidized, supporting a role for redox in chaperone PDI activity (Wang et al., 2013). Reported by several groups, homo-dimerization of the **bb′** domain, regulated by the x-linker region, is suggested to block substrate access, regulating PDI activity (Byrne et al., 2009; Wallis et al., 2009; Bastos-Aristizabal et al., 2014). Molecular dynamic simulation of three-dimensional structures suggested that in solution hPDI may adopt more rigid structures facilitated by inter-domain salt-bridges, encouraging inter-domain disulfide bond formation (Yang et al., 2014). These exciting advances in structural analysis have expanded our knowledge of this highly complex protein and have accelerated our move toward understanding the functional diversity of PDI (The mechanistic details of PDI are summarized in Figure 1).

## Molecular chaperones, PDI, and cellular stress

The cell responds to an increase in immature or misfolded proteins through a concerted protein network response, involving the upregulation of several molecular chaperones that prevent protein aggregation, termed the unfolded protein response (UPR), followed by ER-associated degradation (ERAD), which specifically recognizes terminally misfolded proteins. Molecular chaperones such as the heat shock proteins (e.g., HSP70 and HSP90) are ubiquitously expressed in all cells and in all subcellular compartments. They represent a diverse group of proteins and are characterized based on their dependence on ATP, *holdases*, are ATP dependent and *foldases*, are independent of ATP, the former considered the first line of defense (reviewed by Niforou et al., 2014). Bip/GPR78 and PDI, among other chaperones, function in the ER stress response as neuro-protectors by alleviating the level of misfolded proteins. However, by mechanisms which involve increased cellular stress, these “quality control systems” fail leading to the aggregation and deposition of mis-folded proteins, which not only result in loss of protein function but also lead to cell toxicity and ultimately cell death, a pathway linked to many neurodegenerative diseases.

**Figure 2:**
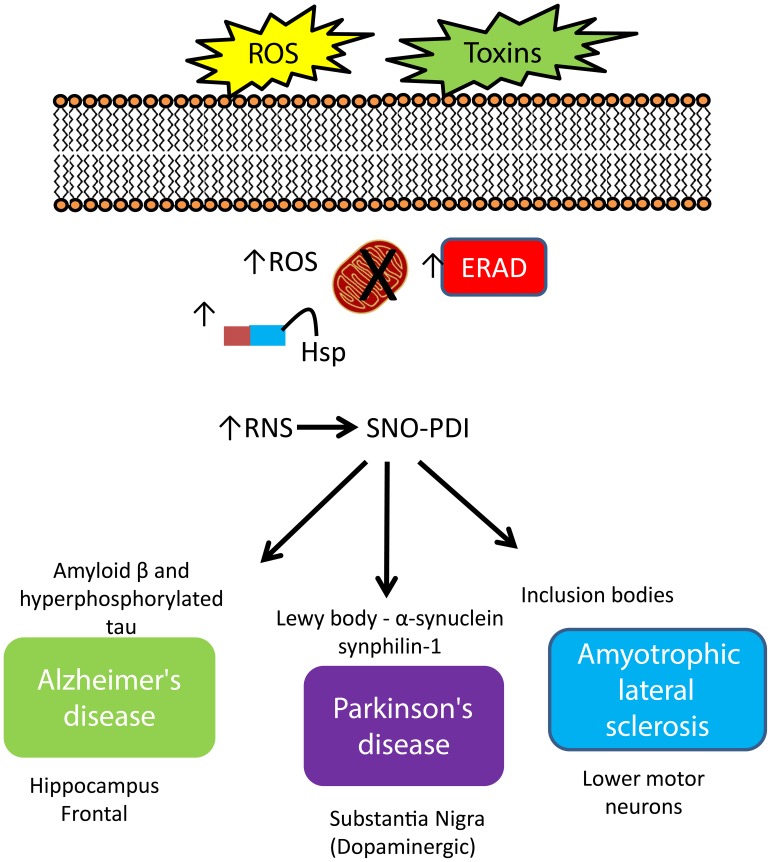
**Increased expression of PDI and regulation of protein misfolding in neurodegenerative disease**. An increase in cellular stress originating from both environmental and intracellular sources initiates a cascade of defenses to protect the cell against damage, including increased expression of molecular chaperones, initiation of the ER-stress response, where the role of PDI strongly features. The response of PDI generates a signature pattern across all neurodegenerative conditions indicating a key role for this protein as a neuroprotector against cellular stress. However, S-nitrosylation impacts activity and compromises this protective role leading to an increase in misfold proteins, creating aggregates, contributing to cell death.

Excess reactive oxygen and nitrogen species (ROS/RNS) generated through oxidative metabolism, or triggered from environmental toxins and also normal aging can cause an imbalance between the production of ROS/RNS and the cell's defense systems, including anti-oxidant enzymes (such as the glutaredoxin system), glutathione, and molecular chaperones (Nakamura and Lipton, 2009). Neurons are particularly vulnerable due to their comparatively low levels of certain antioxidants and reliance on energy from ROS/RNS-generating mitochondrial metabolism (Mattson et al., 2002). Pertinent to this review is the modification of PDI through S-nitrosylation, the addition of a nitric oxide (NO) moiety to a reactive thiol (-SH) group (SNO-PDI). Under normal physiological conditions, S-nitrosylation modulates the function of target proteins, playing a dynamic role in numerous biological processes. In contrast, under pathological conditions, stress induced S-nitrosylation of certain proteins, including PDI, can alter protein function triggering a cascade of events resulting in neuronal cell death that ultimately results in neurodegeneration (Nakamura and Lipton, 2007; Nakamura et al., 2013). Increased PDI expression has been reported in neurodegenerative diseases, to ease ER stress from the accumulation of misfolded proteins, however, the neuroprotective effect of its disulfide-isomerase and chaperone activity can be inhibited by S-nitrosylation (Uehara et al., 2006; Andreu et al., 2012), discussed below with respect to AD, PD, and ALS (summarized in Figure 2).

## SNO-PDI colocalizing with amyloid β and tau: implications in Alzheimer's disease

The key morphology of AD includes cerebral atrophy, β-amyloid (Aβ) deposits, and neuritic changes such as neurofibrillary tangles (NFT) (reviewed in Hardy, 2003), which manifests clinically as a progressive loss in cognition, mood changes, and memory loss (Alzheimer, 1907). These NFTs contain hyperphosphorylated, microtubule-associated tau and are linked to the clinical progressive stages of AD (Braak and Braak, 1991; Morishima-Kawashima and Ihara, 2002). Several metabolic mechanisms also contribute to disease progression, including glutamate toxicity, calcium overload, an increase in cellular stress linked with mitochondrial and ER dysfunction, protein misfolding and a dysregulation of autophagy (Sattler and Tymianski, 2000; Calabrese et al., 2006; Nakamura and Lipton, 2007; Nixon, 2007; Nakamura and Lipton, 2008).

The accumulation of misfolded proteins and influx of cellular Ca^2+^, associated with AD, can cause ER stress in neurons and are considered to contribute to AD (Schroder, 2008). A role for PDI in contributing to AD pathology has been supported through evidence of its co-localization with NFT and the dystrophic neurites of senile plaques in AD patient brain tissues, particularly in the hippocampus and frontal lobe, areas most affected by AD (Honjo et al., 2010, 2012). Co-localization of PDI with tau to the ER was also reported in the SH-SY5Y cell line, a model of AD pathology (Xu et al., 2013). Interestingly, a small number of NFTs immuno-positive for PDI was found in normal control brains, but these were few and scattered (Honjo et al., 2010). However, it may indicate that in normal aging brains ER stress can result in misfolded proteins, where a role for PDI is likely.

In AD, an increase in cellular ROS/RNS is associated with alterations in Aβ metabolism (Dalle-Donne et al., 2006; Mangialasche et al., 2009). These changes in cellular stress also targets susceptible proteins and damages mitochondria (related to Aβ production). ROS-damaged mitochondria results in the oxidation of mitochondrial DNA (genes of oxidative phosphorylation are reduced), lipids [increased 4-hydroxy-2-nonenal (HNE)] (Hardas et al., 2013) and proteins (nitration of enolase) leading to mitochondrial dysfunction and impaired cellular respiration (Castegna et al., 2002). Moreover, mitochondria-derived ROS can also enhance Aβ formation, contributing to cell death (Leuner et al., 2012). Together with the presentation of specific mutations in the presenilin-1 gene, overt mitochondrial damage, where a role for cellular ROS predominates, is considered to factor strongly in the pathogenesis of AD (reviewed in García-Escudero et al., 2013).

Important to this review is the impact of increased cellular ROS on PDI activity and its role in AD. In AD brain an increased expression of PDI was reported, however, pathophysiological relevant amounts of S-nitrosylated PDI was also noted (Uehara et al., 2006; Hoffstrom et al., 2010; Andreu et al., 2012). In these studies, S-nitrosylation of PDI, through the redox active thiols of the **a** and **a′** domain (Figure 1C), inhibited enzyme activity and resulted in the accumulation of poly-ubiquitinated proteins, an increase in ER stress and induction of apoptosis (Uehara et al., 2006; Nakamura and Lipton, 2009). ER stress can initiate a cascade of events including activation of the UPR, which aims to slow down protein synthesis and increase protein folding capacity (reviewed in Salminen et al., 2009). For reasons not entirely understood this system becomes overwhelmed resulting in the increase in protein aggregation, a contributing factor to several neurodegenerative conditions. S-glutathionylation of PDI, induced through RNS, may also factor in this mechanism (Townsend et al., 2009). The accrual of unfolded proteins in the ER lumen is in fact sufficient to produce ROS/RNS, suggesting a vicious cycle of ER stress and local oxidative stress finally leading to cell death when unresolved (Malhotra and Kaufman, 2007). The role of PDI in preventing aggregates, such as Aβ, extends also to NFT, where PDI inhibited nucleation and elongation of Tau_244−372_ fibrillization in SH-SY5Y cells, again illustrating its role as a neuroprotector (Xu et al., 2013).

A homolog of PDI, protein disulfide isomerase P5 (P5), was found to also co-localize with tau in NFTs (Honjo et al., 2014). Knock-down of PDI or P5 in SH-SY5Y cells resulted in decreased cell viability under ER stress (Honjo et al., 2014). Like PDI, the P5 protein was also shown to be S-nitrosylated in AD patient brains, however, unlike that reported for PDI, expression levels of P5 were significantly decreased (Honjo et al., 2014). ERp57, another PDI family protein, has been reported in the CSF of AD patients physically associated with amyloid-β (Erickson et al., 2005), suggesting a potential role as a carrier protein that prevents aggregation of Aβ. Together, these studies clearly indicate a direct association between SNO-PDI, and protein misfolding, understanding of which will identify potential new therapeutic targets for AD.

## α-synuclein aggregation regulated by SNO-PDI in Parkinson's disease

Build-up of protein aggregates strongly features in PD pathology. Here, cytoplasmic Lewy bodies, which stain for aggregated α-synuclein in the substantia nigra, results in the clinical presentation of bradykinesia, rest tremor, rigidity, gait, and postural abnormalities (Spillantini et al., 1997; Baba et al., 1998; Kawamata et al., 2001; Neystat et al., 2002). This is also a heterogeneous disease where protein aggregation and increased cellular stress, among others, contribute to disease pathology (reviewed in Nakamura and Lipton, 2011). Pharmacological models of PD *in vitro* have illustrated that enhanced production of ROS is the main cause of ER stress, where a significant up-regulation of PDI and ERp57 are linked to a global ER stress response (Ryu et al., 2002; Holtz and O'Malley, 2003; Holtz et al., 2006). These findings have also been confirmed in α-synuclein transgenic mouse models, as well as in brain tissue derived from PD patients (Conn et al., 2004; Colla et al., 2012). Although clinically distinct from AD, S-nitrosylated PDI was also reported in PD brain tissue, where S-nitrosylation of PDI inhibited its enzymatic activity, resulting in the accumulation of polyubiquinated proteins, indicating similarities in mechanisms underlying different neurodegenerative conditions (Uehara et al., 2006).

*In vitro* studies have shown that PDI colocalized and interacted with α-synuclein in dopaminergic MES cells, and under normal physiological conditions, forms a complex with α-synuclein, in the process, preventing abnormal aggregation (Honjo et al., 2011a; Xu et al., 2014). However, when PDI was S-nitrosylated, this interaction was disturbed, resulting in α-synuclein oligomerization, indicating a key role for the **a** and **a′** domains in this mechanism (Wu et al., 2014). The authors concluded that PDI can relieve neurodegeneration by decreasing α-synuclein aggregation, but when S-nitrosylated the consequential dysfunction of PDI results in α-synuclein aggregation. Chemical modification of PDI promotes the aggregation and build-up of the minor Parkinsonian-specific biomarker synphilin-1 in a NO dependent manner (Uehara et al., 2006; Uehara, 2007). However, over-expression of wild-type PDI (non-SNO-PDI) reduced synphilin-1-containing aggregates in SHSY-5Y cells (Uehara et al., 2006). A recent paper examining the effect of SNO-PDI formation on α-synuclein aggregation and Lewy-like neurite formation, found that in addition to SNO-PDI formation activating α-synuclein aggregation, it was also seen to provoke co-localization and in parallel the formation of α-synuclein:synphilin-1-containing Lewy-body-like aggregates, supporting the role of SNO-PDI in PD pathogenesis (Kabiraj et al., 2014). Although the functional contribution of PDIs to the pathogenesis of PD *in vivo* requires further validation, collectively these studies illustrate that up-regulation of PDI represents a protective response to abnormal protein aggregation, contributing to the re-establishment of protein homeostasis, whereas S-nitrosylation clearly impacts protein aggregation and deposition.

## SNO-PDI, SOD1, NOX, and amyotrophic lateral sclerosis

ALS is characterized by the accumulation of cytoplasmic aggregated ubiquitinated proteins in motor neurons and surrounding oligodendrocytes, which clinically presents as progressive muscle weakness, typically with a 3–5 year survival (reviewed in Blokuis et al., 2013). Mutations in 16 genes have been identified to be associated with classical ALS, including mutations in superoxide dismutase (SOD1), TARDBP, fused in sarcoma (FUS), UBQLN2, profiling, and C9ORF72, but these are rare (Ferraiuolo et al., 2011; Rademakers and Blitterswijk, 2012; Chen et al., 2013). Approximately 90% of all ALS cases are sporadic, with the second most frequent, familial ALS, attributed to dominantly inherited mutations in Cu/Zn SOD1 (Rosen, 1993). The major inclusions associated with both familial and sporadic ALS are the TDP-43-positive ubiquitinated protein aggregates that accumulate in the cytoplasm, where PDI was also shown to reside (Honjo et al., 2011b). Another inclusion associated with ALS, FUS is normally located in the nucleus but in ALS is redistributed to the cytoplasm (Dormann et al., 2010). In NSC-34 cells, mutant FUS expression triggers ER stress and co-localizes with PDI (Farg et al., 2012). Moreover, PDI was shown to co-localize with FUS-positive ubiquitinated inclusions, in human ALS patient spinal cords, supporting the *in vitro* cell models.

A significant increase in PDI expression, alongside other ER stress markers such as ERp57, was reported in ALS spinal cord and CSF compared to controls and also in the G93A SOD1 mouse model of ALS (Atkin et al., 2006, 2008; Massignan et al., 2007; Honjo et al., 2011a). This increase in PDI expression is not just limited to neurons, but has also been observed in the microglia and astrocytes of murine models (Jaronen et al., 2013). However, in ALS, PDI is also S-nitrosylated impacting its neuroprotective role, again sharing common mechanistic traits to AD and PD (Walker et al., 2010; Jeon et al., 2014). In ALS mouse and cell models, overexpression of PDI decreases both the accumulation of SOD1 aggregates and neuronal cell death, whereas inhibition of PDI using bacitracin or siRNA knockdown increases the formation of aggregates (Atkin et al., 2006, 2008; Walker et al., 2010; Jeon et al., 2014). Furthermore, when production of NO generation via NOS was blocked using N-nitro-L-arginine, the formation of SNO-PDI and mutant SOD1 aggregates significantly decreased (Chen et al., 2013), but interestingly studies using the PDI redox-mutant suggest that it is the chaperone activity of PDI that was responsible (Jeon et al., 2014). The therapeutic effects of Reticulon-4A, found to have a protective role against disease onset and progression in an ALS mouse model, were associated with intracellular redistribution of PDI, supporting the role of PDI as a therapeutic target (Yang et al., 2009).

*In vivo* ALS studies have indicated that NADPH oxidase (NOX) activation and superoxide production are elevated in microglia and may contribute to motorneuron death, through increased cellular S-nitrosylation (Wu et al., 2006). *In vitro* studies showed that PDI acts as an important regulator of NOX activation and subsequent ROS production in microglial cells, suggesting that an up-regulation of PDI may not just have a protective role in protein aggregation but also in regulating UPR-triggered NOX activation, in a cell-type specific manner (Jaronen et al., 2013). Proteomic screening identified PDI and ERp57 as biomarkers for ALS, with ERp57 highlighted for its potential to monitor disease progression (Nardo et al., 2011). Due to the key role PDI appears to play in ALS, a genome association study examined the effects of two specific single-nucleotide polymorphisms (SNPs) in the P4HB gene, which codes for PDI, which showed a significant association with familial ALS, suggesting that P4HB is a modifier gene in ALS susceptibility and may present a potential therapeutic target (Kwok et al., 2013). The same study also identified two genotypes and one diplotype that modify disease survival. These findings are consistent with the view that PDI expression is clearly linked to ALS pathology and either de-nitrosylation of PDI or up-regulation of total PDI may provide a therapeutic benefit in ALS.

## Conclusion

An increase in the generation of ROS/RNS through external and internal cellular mechanisms can overwhelm the anti-oxidant and chaperone systems contributing to an increase in cellular calcium, mitochondrial dysfunction, protein misfolding/aggregation leading to cell death. This pathogenic pathway shares common features across all neurodegenerative conditions despite clear genetic links that differentiate between them. A role for PDI in protein misfolding has emerged as a key link to understanding how these aggregates accumulate in the brain with similar patterns of colocalization of PDI with disease specific aggregates. Despite clear evidence that protein aggregation in central to the pathology of neurodegenerative conditions, many questions remain unanswered. How do these models of protein misfolding translate *in vivo*? Is it possible to generate a therapeutic translation to mimic increased PDI expression that is not subject to S-nitrosylation? Or will increased expression of PDI stimulate unfavorable metabolic reactions, attenuating its potential role as a target therapeutic.

### Conflict of interest statement

The authors declare that the research was conducted in the absence of any commercial or financial relationships that could be construed as a potential conflict of interest.
